# Identification of serum biomarkers for active pulmonary tuberculosis using a targeted metabolomics approach

**DOI:** 10.1038/s41598-020-60669-0

**Published:** 2020-03-02

**Authors:** Yonggeun Cho, Youngmok Park, Bora Sim, Jungho Kim, Hyejon Lee, Sang-Nae Cho, Young Ae Kang, Sang-Guk Lee

**Affiliations:** 1 Department of Laboratory Medicine, Severance Hospital, Yonsei University College of Medicine, Seoul, Republic of Korea; 2 Division of Pulmonology, Department of Internal Medicine, Severance Hospital, Yonsei University College of Medicine, Seoul, Republic of Korea; 3 Institute of Immunology and Immunological Diseases, Yonsei University College of Medicine, Seoul, Republic of Korea; 4 Department of Clinical Vaccine Research, International Tuberculosis Research Center, Seoul, Republic of Korea; 5 Department of Clinical Laboratory Science, College of Health Sciences, Catholic University of Pusan, Busan, Republic of Korea

**Keywords:** Diagnostic markers, Tuberculosis

## Abstract

Although tuberculosis (TB) is a severe health problem worldwide, the current diagnostic methods are far from optimal. Metabolomics is increasingly being used in the study of infectious diseases. We performed metabolome profiling to identify potential biomarkers in patients with active TB. Serum samples from 21 patients with active pulmonary TB, 20 subjects with latent TB infection (LTBI), and 28 healthy controls were analyzed using liquid chromatography-tandem mass spectrometry (LC-MS/MS) followed by multivariate and univariate analyses. Metabolic profiles indicated higher serum levels of glutamate, sulfoxy methionine, and aspartate and lower serum levels of glutamine, methionine, and asparagine in active TB patients than in LTBI subjects or healthy controls. The ratios between metabolically related partners (glutamate/glutamine, sulfoxy methionine/methionine, and aspartate/asparagine) were also elevated in the active TB group. There was no significant difference in the serum concentration of these metabolites according to the disease extent or risk of relapse in active TB patients. Novel serum biomarkers such as glutamate, sulfoxy methionine, aspartate, glutamine, methionine, and asparagine are potentially useful for adjunctive, rapid, and noninvasive pulmonary TB diagnosis.

## Introduction

According to the World Health Organization report, Tuberculosis (TB) caused approximately 10.0 million patients and 1.6 million deaths globally in 2017^1^. Timely diagnosis and treatment are critical in controlling TB successfully. Currently, TB exposure is evaluated with two methods: a tuberculin skin test (TST) and an interferon-gamma release assay (IGRA). However, existing diagnostic techniques cannot distinguish latent tuberculosis infection (LTBI) from active TB because they indirectly identify TB infection through detection of the immunologic reaction to the *Mycobacterium tuberculosis* (Mtb) antigen^2^. Therefore, the identification of blood markers representing the actual TB bacterial presence/burden in the human body is needed to tailor treatment options for patients with positive TST or IGRA results.

Recently, metabolomics has emerged as a potential tool making remarkable progress in novel biomarker research. It can execute multiplexed profiling and compare multiple metabolites in a biological sample^3^. It aims the comprehensive measurement of the metabolites; therefore, highly specialized analytical techniques in combination with sophisticated statistical, mathematical, and bioinformatics analyses are required^4^. Accordingly, metabolomics technologies have been widely utilized in various diseases to screen novel biomarkers, understand the biologic processes and metabolic pathways, and comprehend the response of the host, which enables researchers to develop improved diagnostic tools and treatment strategies^5,6^.

Several metabolomics studies used sputum^7,8^, blood^9–14^, breath^15,16^, and urine^17^ to identify new markers of TB infection or treatment response. However, the number of studies performed remains limited. Based on the previous works, we hypothesized that we could find specific serum metabolites to diagnose patients with active pulmonary TB.

In the present study, we used liquid chromatography-tandem mass spectrometry (LC-MS/MS) to profile the metabolites found in the serum of active TB patients, LTBI subjects, and healthy controls. We conducted multivariate analyses and found that the metabolites measured in this study could accurately discriminate patients with active TB from healthy controls and those with LTBI. We then selected the key metabolites responsible for the distinction and assessed their diagnostic performance in diagnosing patients with active TB. Finally, we examined the correlation between some of the clinical features of TB and the serum levels of the selected metabolites.

## Results

### Subject characteristics

A total of 69 subjects participated in this study (Table 1). There were 21 patients with active pulmonary TB (age, 27 [range 20–50] years; males, n = 10 [47.6%]), 20 subjects with LTBI (age 48.5 [range 23–69] years; males, n = 6 [30.0%]), and 28 healthy controls (age 28 [range 22–57] years; males, n = 10 [35.7%]). There were no differences in baseline characteristics among the three groups except for in age and body mass index. Additionally, there were no differences between the low-risk of relapse group and the moderate-to-high-risk of relapse group among the active pulmonary TB patients (Table 2).

**Table 1:** Clinical characteristics of the subjects^a^.

	Active TB group (N = 21)	LTBI group (N = 20)	Healthy controls (N = 28)	P-value
Age, years, median (range)	27 (20–50)	48.5 (23–69)	28 (22–57)	<0.001^c^
Sex, male	10 (47.6)	6 (30.0)	10 (35.7)	0.518^d^
BMI, kg/m^2^, median (IQR)	20.0 (18.6–21.9)	21.7 (20.9–23.9)	22.1 (20.6–23.4)	0.017^c^
Past TB history	0 (0)	2 (10.0)	0 (0)	0.081^d^
Presence of BCG scar	16 (76.2)	17 (85.0)	19 (67.9)	0.441^d^
Co-morbidities				
Hypertension	0 (0)	2 (10.0)	0 (0)	0.081^d^
Diabetes mellitus	0 (0)	1 (5.0)	0 (0)	0.290^d^
Other^b^	1 (4.8)	3 (15.0)	0 (0)	0.064^d^
Pulmonary TB diagnosis				
Sputum AFB smear, positive	1 (4.8)			
Sputum AFB culture, positive	19 (90.5)			
Extent of chest field^e^				
Less than one-third	17 (81.0)			
Less than two-thirds	4 (19.0)			
More than two-thirds	0 (0)			
Combined extrapulmonary lesion	2 (9.5)			
TST positive	2 (100)	16 (80.0)	0 (0)	<0.001^d^
TST induration, mm, median (range)	15 (12–18)	14 (0–25)	0 (0–4)	<0.001^c^
QFT-GIT positive	19 (100)	20 (100)	0 (0)	<0.001^d^

^a^Data are presented as numbers (percentages) unless otherwise indicated.

^b^Active TB group: B-viral hepatitis, LTBI group: asthma or chronic bronchitis.

^c^P-values were calculated using Kruskal-Wallis tests.

^d^P-values were calculated using Fisher’s exact tests.

^e^TB extent of the unilateral lung was evaluated by chest radiography or chest computed tomography.

Abbreviations: TB, tuberculosis; LTBI, latent tuberculosis infection; BMI, body mass index; IQR, interquartile range; BCG, Bacillus Calmette-Guerin; AFB, acid-fast bacillus; TST, tuberculin skin test; QFT-GIT, QuantiFERON-TB Gold-in-Tube Test.

**Table 2:** Clinical characteristics of the active pulmonary TB group^a^.

	Total (N = 21)	Low-risk (N = 15)	Moderate / High-risk (N = 6)	P-value
Age, years, median (range)	27 (20–50)	26 (20–37)	33 (24–50)	0.205^c^
Gender, male	10 (47.6)	8 (53.3)	3 (50.0)	1.000^d^
BMI, kg/m^2^, median (range)	20.0 (18.6–21.9)	19.6 (17.1–24.6)	20.9 (17.8–25.1)	0.733^c^
Presence of BCG scar	16 (76.2)	12 (80.0)	4 (66.7)	0.598^d^
Comorbidities^b^	1 (4.8)	1 (6.6)	0 (0)	1.000^d^
Pulmonary TB diagnosis				
Sputum AFB smear, positive	1 (4.8)	0 (0)	1 (16.7)	0.286^d^
Sputum AFB culture, positive	19 (90.5)	13 (86.7)	6 (100.0)	1.000^d^
Extent of chest field^e^				
Less than one-third	17 (81.0)	12 (80.0)	5 (83.3)	1.000^d^
Less than two-thirds	4 (19.0)	3 (20.0)	1 (16.7)	
Combined extrapulmonary lesion	2 (9.5)	2 (13.3)	0 (0)	1.000^d^

^a^Data are presented as numbers (percentages) unless otherwise indicated.

^b^B-viral hepatitis.

^c^P-values were calculated with Mann-Whitney U tests.

^d^P-values were calculated with Fisher’s exact tests.

^e^TB extent of the unilateral lung was evaluated by chest radiography or chest computed tomography.

Abbreviations: TB, tuberculosis; BMI, body mass index; BCG, Bacille Calmette-Guerin; AFB, acid-fast bacillus.

### Multivariate analysis

**Figure 1:**
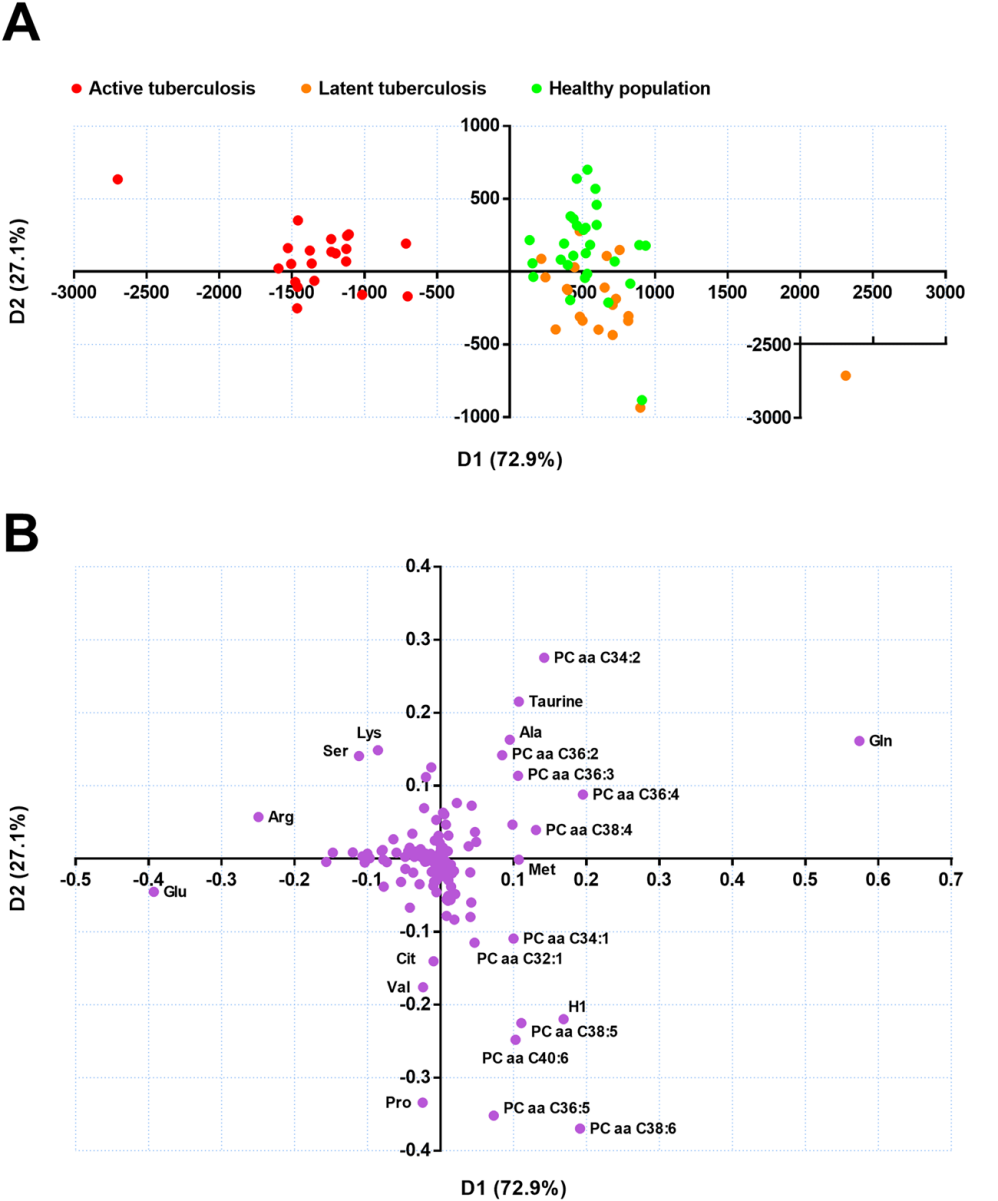
Principal component analysis-discriminant analysis (PCA-DA) score and loading plot of the metabolites measured in patients with active tuberculosis, participants with latent tuberculosis, and healthy controls. (**A**) In the score plot, each dot represents an individual and is colored in accordance with the embedded legends. (**B**) Loadings of the two principal components (D1 and D2) are depicted. The metabolites located distant from the origin are labeled with chemical names.

Principal component analysis (PCA) with the data from the 69 samples depicted that patients with active TB could be easily distinguished from the others, as shown in the score plot (Supplementary Fig. S1). A similar distinction of the cluster was reproduced in the principal component analysis-discriminant analysis (PCA-DA, Fig. 1A). Meanwhile, participants with LTBI could not be distinguished from healthy controls in either multivariate analysis. Among the analyses, in contrast to the PCA, the PCA-DA is supervised by the predefined response, i.e., population groups according to the disease status. Thus, it is more plausible to interpret the results in reverse order of the analyses performed. The reliability of the separation pattern inferred by the PCA-DA is cross-validated, as the pattern was also made in an unsupervised analysis of PCA. The loading plot of the PCA-DA indicated that the variables most responsible for the distinction of the active tuberculosis group from the other groups, in which separation was made along the D1 axis of the plots, were increased glutamate (Glu) and decreased glutamine (Gln; Fig. 1B).

### Univariate analysis

The fold change in mean metabolite concentration in patients with active TB over that in the other groups was calculated using the false discovery rate adjusted p-value (q-value) and is presented in a volcano plot (Supplementary Fig. S2). The most significant change determined by the q-value was the decrease in Gln concentration (Supplementary Fig. S2 and Supplementary Table S1) in patients with active TB. In addition, an increase in the Glu level also belonged to the variables showing significant changes, as determined by the arbitrary cutoff values for fold change and the q-value used to depict significant variables (Supplementary Fig. S2 and Supplementary Table S1). In combination with the results from the multivariate analyses, we concluded that the reciprocal change in the Glu and Gln concentration was the most significant factor that differentiated patients with active TB from the other groups.

**Figure 2:**
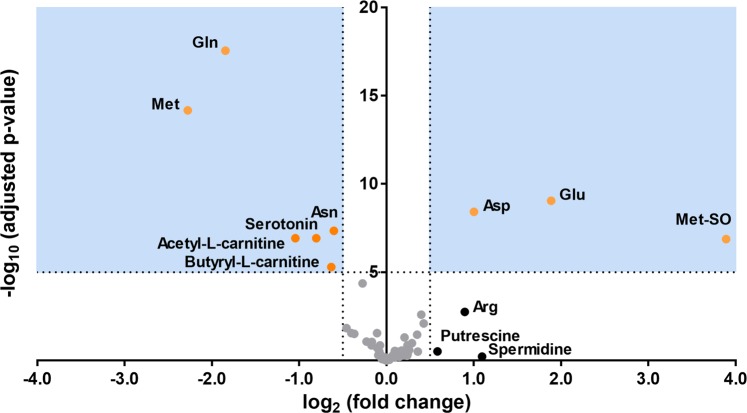
Volcano plot of the 76 non-lipid metabolites (amino acids, biogenic amines, acyl-carnitines, and hexose) derived from the comparison between patients with active tuberculosis and those in the other groups (i.e., patients with latent tuberculosis and healthy controls). The p-values are adjusted for the false discovery rate against the log_2_ fold changes. For each metabolite, the log_2_ fold change is displayed on the x-axis and the -log10 (p-value adjusted for false discovery rate) is displayed on the y-axis. Cutoff points for the p-value (<0.00001; −log_10_ (0.00001) = 5) or fold change (> 1.414; log_2_ (1.414) = 0.5 or <0.707; log_2_ (0.707) = −0.5) are indicated with dotted lines. The areas fulfilling both criteria are colored in blue, and the metabolites belonging to the area are shown as orange dots. Metabolites fulfilling only one criterion are shown as black dots; the others are colored in grey. Abbreviations: Glu, glutamate; Gln, glutamine; Met, methionine; MetSO, sulfoxy methionine; Asp, aspartate; Asn, asparagine; Arg, arginine.

Among 104 lipid metabolites (lysophosphatidylcholines, phosphatidylcholines, and sphingomyelins), 46 lipid metabolites were significantly changed in patients with active TB compared to the others (Supplementary Table S1). We presented diagnostic performances of 12 representative lipid metabolites consisting of 7 phospholipids and 5 lysophospholipids (as the order of least q-values, Supplementary Fig. S3 and S4). However, our lack of understanding of the metabolic pathways in which these lipid metabolites are involved restricted further reasoning. Thus, we conducted another univariate analysis using 76 non-lipid metabolites (amino acids, biogenic amines, acyl-carnitines, and hexose). In the new analysis using simplified data, the reciprocal changes in Glu and Gln were also highly ranked on the list of significant variables (Supplementary Table S2). Notably, reciprocal changes in the other metabolically related metabolites were also found. Methionine sulfoxide (MetSO) increased as methionine (Met) decreased, and aspartate (Asp) increased as asparagine (Asn) decreased (Fig. 2 and Supplementary Table S2).

### Diagnostic utility of selected metabolites

**Figure 3:**
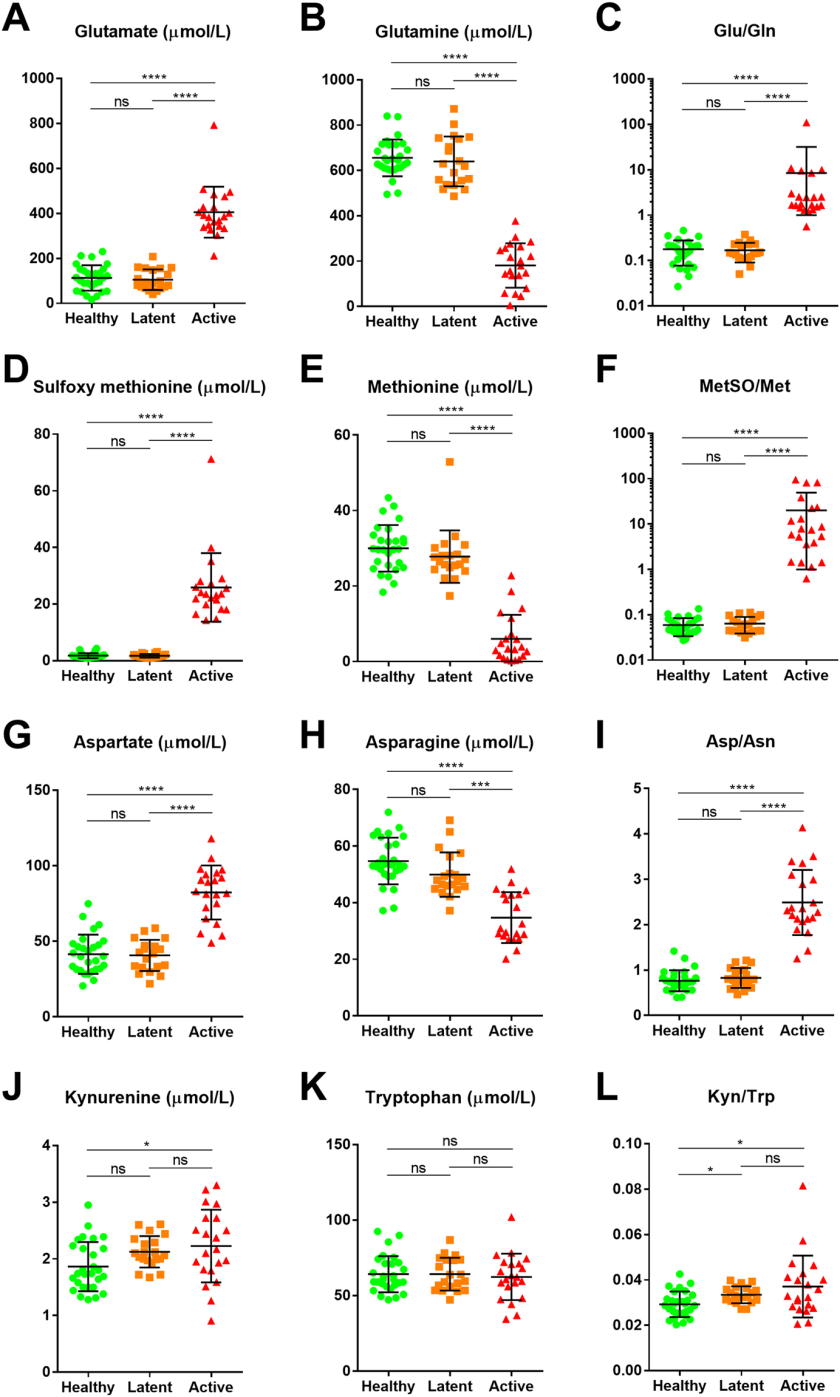
The levels or ratios of selected metabolites among study populations. The median values of selected metabolites and their ratios were compared among the healthy population, patients with latent tuberculosis, and patients with active tuberculosis using Kruskal-Wallis tests. Note that the figures representing Glu/Gln and MetSO/Met ratio are in a logarithmic scale (C and F), while the others are on a linear scale. Abbreviations: Glu, glutamate; Gln, glutamine; Met, methionine; MetSO, sulfoxy methionine; Asp, aspartate; Asn, asparagine; Kyn, kynurenine; Trp, tryptophan.

**Figure 4:**
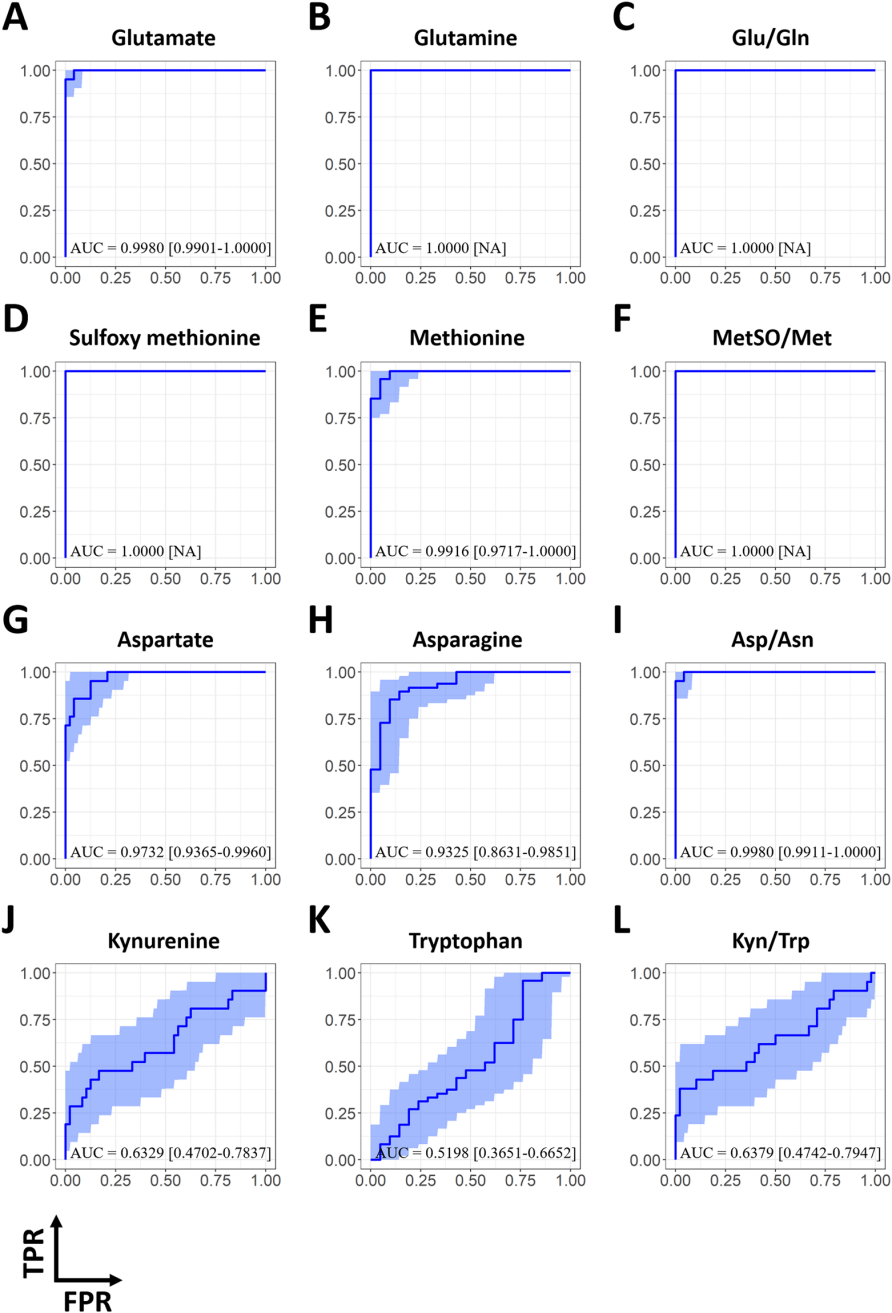
ROC curves of the selected metabolites and their ratios for the differentiation of patients with active tuberculosis from those in the other groups, including patients with latent tuberculosis infection and healthy controls. Blue lines indicate ROC curves and royal blue regions show the 95% CI of the curve computed with 2000 bootstrap replicates. The AUC, followed by its 95% CI in brackets, were also denoted over the bottom side of each figure. Abbreviations: TPR, true positive rate; FPR, false positive rate; Glu, glutamate; Gln, glutamine; Met, methionine; MetSO, sulfoxy methionine; Asp, aspartate; Asn, asparagine; Kyn, kynurenine; Trp, tryptophan; ROC, receiver operating characteristic; CI, confidence interval; AUC, area under the curve.

The diagnostic values of Glu, Gln, MetSO, Met, Asp, Asn, and the ratios between the metabolically related partners (i.e., Glu/Gln, MetSO/Met, and Asp/Asn) were assessed in patients with active TB. Additionally, the utilities of kynurenine (Kyn), tryptophan (Trp), and their ratio, Kyn/Trp, were evaluated because the activation of indoleamine 2,3-dioxygenase (IDO-1), which converts Trp to Kyn, has been widely reported in macrophages exposed to Mtb^18–20^. When comparing the metabolites’ median values or ratios amongst the three populations, patients with active TB showed significantly elevated or decreased levels in most metabolites (except for Trp) when compared to either participants with LTBI or the healthy controls (Fig. 3). Similar patterns were found when the levels of variables were compared between patients with active TB and the other populations combined, while the differences in Kyn, Trp, and Kyn/Trp were insignificant (Table 3). In both comparisons, the ratio of each metabolically relevant metabolite (Glu/Gln, MetSO/Met, and Asp/Asn) showed equal or smaller p-values than for each metabolite alone. In addition, the area under the curve (AUC) in the receiver operating curve (ROC) analysis for each Glu, Glu, MetSO, Met, Asp, Asn, Glu/Gln, MetSO/Met, and Asp/Asn exceeded 0.9, indicating good clinical performance in the ability to diagnose patients with active TB (Fig. 4). Meanwhile, the clinical performance of Kyn, Trp, and Kyn/Trp were poor, as the 95% confidence interval (CI) of their AUC spanned 0.5 (Table 3). The AUC values of the ratios (Glu/Gln, MetSO/Met, and Asp/Asn) were equal to or higher than those of the corresponding metabolite (Table 3). Further, we aimed to test whether the variables could be used to stratify patients with active TB according to their degree of risk burden (Supplementary Fig. S5) or extent of pulmonary involvement (Supplementary Fig. S6), but none of the variables showed a significant difference among the subpopulations of patients with active TB.

**Table 3:** Comparison of the median values and diagnostic utility of selected metabolites and ratios in diagnosing patients with active tuberculosis.

Metabolites and ratios	Active TB^a^	The others^a^	P-value^b^	AUC^c^
	(N = 21)	(N = 48)		
Glu (μmol/L)	386.0 [344.0–428.0]	100.2 [70.9–150.0]	<0.001	0.9980 [0.9901–1.0000]
Gln (μmol/L)	180.0 [136.0–257.0]	638.0 [596.0–715.0]	<0.001	1.0000 [NA]
Glu/Gln	1.7650 [1.5341–2.9930]	0.1506 [0.1133–0.2144]	<0.001	1.0000 [NA]
MetSO (μmol/L)	23.5 [19.8–27.2]	1.5 [1.2–2.0]	<0.001	1.0000 [NA]
Met (μmol/L)	3.4 [1.5–7.4]	28.3 [24.9–31.9]	<0.001	0.9916 [0.9717–1.0000]
MetSO/Met	7.4359 [3.5374–22.3197]	0.0516 [0.0424–0.0763]	<0.001	1.0000 [NA]
Asp (μmol/L)	83.2 [72.4–94.2]	41.1 [31.8–47.4]	<0.001	0.9732 [0.9365–0.9960]
Asn (μmol/L)	30.9 [28.3–42.9]	51.8 [46.5–58.5]	<0.001	0.9325 [0.8631–0.9851]
Asp/Asn	2.3108 [2.1264–2.9890]	0.7557 [0.6119–0.9315]	<0.001	0.9980 [0.9911–1.0000]
Kyn (μmol/L)	2.2 [1.8–2.7]	2.0 [1.7–2.3]	0.082	0.6329 [0.4719–0.7940]
Trp (μmol/L)	62.4 [56.0–71.1]	60.7 [56.1–73.5]	0.804	0.5198 [0.3651–0.6652]
Kyn/Trp	0.0329 [0.0282–0.0412]	0.0309 [0.0271–0.0350]	0.071	0.6379 [0.4742–0.7947]

^a^Median values are presented with interquartile ranges. ^b^P-values were calculated using Mann-Whitney U tests. ^c^The AUCs of receiver characteristic analysis to predict patients with active TB were presented with their 95% confidence interval (CI) computed by 2000 bootstrap replicates. For the metabolite or ratio with the AUC value of 1, CI was not presented to avoid misunderstanding.

Abbreviations: Glu, glutamate; Gln, glutamine; Met, methionine; MetSO, sulfoxy methionine; Asp, aspartate; Asn, asparagine; Kyn, kynurenine; Trp, tryptophan; AUC, area under the curve; NA, not available.

## Discussion

This study demonstrated that levels of serum metabolites and their ratios could differentiate adults with active pulmonary TB from both adults with LTBI or adults with no TB infection in South Korea, an area with an intermediate burden of TB and low burden of human immunodeficiency virus infection. The distinguishing metabolites included Glu, Gln, MetSO, Met, Asp, Asn, and the ratios between the metabolically related partners (Glu/Gln, MetSO/Met, and Asp/Asn). In addition, we attempted to determine the role of the metabolites along with the burden and risk of relapse in pulmonary TB.

Previous metabolomics studies that have searched for markers in serum or plasma samples to distinguish patients with active TB from others have found a variety of candidate metabolites with the help of mass-spectrometry^11,13,14,21^ or nuclear magnetic resonance spectroscopy^12,22^. As the experiments in the previous studies were conducted in a non-targeted manner, the types of biosignatures were diverse, ranging from modified lipids and peptides to simple amino acids. In this study, we used mass-spectrometry in a targeted manner using a commercial metabolite library kit. Further, in an analysis of diagnostic performance, we emphasized the non-lipid metabolites from the whole repertoire of metabolites presented by the kit, for the ease of deducing mostly altered ‘metabolic pathway’ and thereby getting robust derivative markers representing the pathway, expectedly, superior to each metabolite marker itself. These differences in methodology resulted in a limitation in the number and kinds of metabolites assessed in the current study compared with those of previous studies.

The major amino acid changes we discovered (Figs. 1 and 2) were in line with the majority of the findings from the previous studies that were conducted in a non-targeted manner. Frediani *et al*.^11^ and Zhou *et al*.^12^ found elevated Glu levels in active TB patients, and Weiner *et al*.^13^ found low levels of Gln and high levels of Asp in active TB patients in comparison with LTBI subjects and healthy controls. Concordantly, Sun *et al*.^22^ reported a decrease in Gln in active TB patients in comparison with non-TB controls, which consisted of patients with respiratory tract infection and healthy children. In line with its diagnostic performance, a recent study by Weiner *et al*.^21^ demonstrated that a decrease in Gln constituted one of the prognostic biosignatures that identified individuals who progress to clinical TB among patients with preclinical TB. Of note, Weiner *et al*.^13^ reported that the Gln/Glu ratio in active TB patients was significantly lower than in LTBI or healthy controls; however, the ratio values were not presented. As our study was conducted in a targeted manner using calibrators for each metabolite, we might have measured each metabolite more precisely and obtained a more reliable numerical relationship between the measured values than those in experiments conducted in a non-targeted manner. Including Glu/Gln, we found that the ratios between the metabolically related partners among distinguishing metabolites showed similar or superior diagnostic utility over the values of each metabolite alone (Fig. 3 and 4). These findings collectively support the idea of amino acids and their ratios as diagnostic markers of active TB.

Mtb not only produces specific virulence factors that cause disease^23^, but also adapts to resist the stresses encountered inside the host^24^, including hypoxia within granulomatous lesions^25^, low pH, reactive oxygen and reactive nitrogen molecules produced by the host, and lack of nutrients inside macrophages^26^. The bacterium is a facultative intracellular pathogen that thrives inside the phagosome; therefore, understanding the process of nutrient acquisition and its metabolic pathways is essential.

Nitrogen is a fundamental building block of biomolecules such as amino acids, nucleotides, cell wall components, and organic cofactors. Bacteria use ammonium (NH4^+^), which is first incorporated into Glu and Gln and works as a primary nitrogen donor, as the key molecule for central nitrogen metabolism^27^. Lee *et al*. found that Mtb converts Gln to Glu using glutamate synthase to neutralize cytoplasmic pH in the host environment, which makes it a successful intracellular pathogen^28^. Maintenance of the Glu pool is also linked to ergothioneine, which plays a critical role in the redox homeostasis of Mtb^29^. In our study, the active pulmonary TB patients showed high Glu and low Gln levels in comparison with the LTBI group and healthy controls, which is consistent with the adaptive mechanism of Mtb.

During infection, Mtb markedly expresses the Asn transporter to capture Asn from macrophages; additionally, it expresses asparaginase to hydrolyze Asn into Asp and ammonia^30^. Asn is one of the amino acids essential for Mtb to resist acidic circumstances in the phagosome by serving as a substrate for the production of the weak base ammonium. Additionally, the absence of Asn may affect T cell activation and differentiation^31^. The pathogen could suppress the immune reaction of the host by inducing starvation of Asn in host cells.

As an immune response to Mtb infection, the macrophage undergoes a respiratory burst and produces high levels of reactive oxygen species to resist and kill the mycobacteria^32^. MetSO is an oxidation product of Met with reactive oxygen species^33^ and is known to be a biomarker of oxidative stress in various human conditions and diseases^34,35^. Our results showed that the levels of MetSO were increased in the active TB group, which reflects the host response to Mtb infection.

According to previous studies, IDO-1, an immunoregulatory enzyme that breaks down Trp to Kyn, has been studied as a prognostic marker of pulmonary TB^18–20^. The activity of IDO-1 is usually estimated by the serum Kyn-to-Trp ratio and is increased in TB patients. However, we could not detect meaningful differences in the levels of Trp, Kyn, or the Kyn/TRP ratio (Table 3).

To validate classification performances of biosignatures of the present study, external validation on a publicly available dataset by Weiner *et al*.^13^ was conducted. To our surprise, the difference in the levels of selected amino acids between active TB and the others was less obvious in their study population (Supplementary Fig. S7). Accordingly, the diagnostic performance of the selected amino acids (Glu, Gln, Asp, Asn, and Met) in diagnosing patients with active TB was diminished compared to those in our study population (Supplementary Fig. S8). Besides, the ratio of metabolically related partners (Glu/Gln and Asp/Asn) gave little benefit in the diagnosis. Meanwhile, changes in kynurenine and tryptophan were more evident in their study population than in ours, resulting in superior diagnostic performances, and the calculation of their ratio (Kyn/Trp) gave apparent additive effect (Supplementary Fig. S7 and Supplementary Fig. S8). Collectively, these findings support the idea of finding a derivative marker representing changes in particular metabolic pathways gives superior diagnostic performance over each marker itself, while the representative derivative markers, and presumably underlying mostly altered pathway, differed in the study by Weiner *et al*. and in ours.

To address this issue, we assumed that differences between the two studies in certain clinical features of patients with active TB resulted in different metabolic pathways that were changed in patients with active TB. Besides its utility as a prognostic marker in pulmonary TB^18–20^, the ratio of Kyn/Trp has been reported in various inflammatory conditions^36^ including sepsis due to other bacteria^37^. When the signs and symptoms in patients with active TB were compared, those in our study showed a low rate of sputum acid-fast bacillus (AFB) smear positivity (1/21; Table 1 and Table 2) and only two-thirds had a cough (13/21; data not shown), while all subjects in Weiner *et al*.’s study had sputum AFB smear positivity, had cough for more than two weeks, and had at least two additional symptoms^13^. Thus, it is plausible to hypothesize that the degree of systemic inflammation might be more severe in the active TB patients in Weiner *et al*. study compared to that in our study, and consequently more prone to the alteration of metabolic pathways that general host inflammation is related rather than the specific metabolic pathways that TB biology is involved. To test our hypothesis, future metabolomic studies of patients with active TB with diverse systemic inflammation statuses are needed.

There were some limitations to this study. First, this was a cross-sectional study. The serum samples were obtained only once within the first week of drug therapy. Second, there were no differences in the levels of the serum biomarkers between the LTBI group and the healthy controls. This may have been due to the burden of Mtb in the LTBI group being insufficient to show a difference in the levels of serum metabolite. Third, we could not determine the role of these metabolites in active TB patients when we stratified by risk level or disease extent. Due to the relatively small numbers of study subjects, only six participants were enrolled in the moderate-to-high-risk group of the active TB population; this sample size was likely too small to reveal differences within active TB patients (Supplementary Fig. S5, Supplementary Fig. S6, and Table 2). Future studies with larger sample sizes are needed. Finally, due to the lack of our ability to interpret the complex nature of metabolism between diverse analytes, we focused on the analysis of diagnostic performance to the 76 non-lipid metabolites. Although this subset contained the metabolites showing the most significant change, information from the remaining 111 lipids was also presented. Since numerous lipid metabolites in our study showed good diagnostic performances by themselves (Supplementary Fig. S3 and Supplementary Fig. S4), further research into the alterations in lipid metabolism by Mtb is needed. The complete list of metabolites showing significant changes in volcano plots are presented (Supplementary Table S1 and Supplementary Fig. S2, respectively), and we made the raw data publicly available.

In summary, the metabolic adaptation strategies of Mtb to survive the intracellular environment and host responses during TB infection might be used in the early diagnosis of active pulmonary TB. The novel serum biomarkers of Glu, Gln, MetSO, Met, Asp, Asn, and their ratios are potentially useful for adjunctive rapid and noninvasive pulmonary TB diagnosis.

## Methods

### Study population

We collected clinical information and specimens from participants aged 20 years and older. Participants were recruited for two clinical studies: one recruited participants between November 2010 and March 2012, and the other study recruited participants between May 2015 and June 2016 from Severance Hospital, which is a tertiary referral hospital in South Korea. Individuals with human immunodeficiency virus infection, end-stage renal disease, malignant diseases, and those who had received anti-TB therapy for more than two weeks or immunosuppressive therapy within three months of enrollment were excluded from the study. The Institutional Review Board of Severance Hospital approved the study protocol (#4-2010-0527 and #4-2014-1108). All study subjects provided written informed consent, and the study was carried out in accordance with the relevant guidelines and regulations. The mycobacterium culture and AFB smear of sputum specimens for TB were conducted in biosafety cabinet class 2, complying with local and national regulations on containment equipment.

We classified study subjects into an active pulmonary TB group, a LTBI group, and a healthy control group. Patients with active pulmonary TB were diagnosed based on clinical, radiological, microbiological, and pathological data. A positive Mtb culture result from respiratory specimens or the presence of caseating granulomas in lung tissue was used to confirm the diagnosis of active pulmonary TB. Based on the risk factors for relapse, i.e., the presence of a cavitary lesion on chest imaging and positive sputum culture after two months of anti-TB treatment, we subdivided the active pulmonary TB patients into three groups. The low-risk group did not have any risk factors, the moderate-risk group had one risk factor, and the high-risk group had both risk factors. We also classified active TB patients into three groups based on the extent of the chest lesion, which was determined by the proportion of the affected chest field from chest radiograph or chest computed tomography (CT).

Participants with LTBI fulfilled the following criteria: (i) no history of TB; (ii) no suggestive symptoms of TB; (iii) recent contact with active pulmonary TB patients; and (iv) a positive result from the QuantiFERON-TB Gold-in-Tube Test (QFT-GIT; Qiagen, Hilden, Germany). Healthy controls were defined as those who met criteria (i) and (ii) but had no recent contact with active pulmonary TB patients and had a negative result on the QFT-GIT test.

### Quantitative metabolome analysis

A targeted quantitative approach using a combined direct flow injection-tandem mass spectrometry (FIA-MS/MS) and LC-MS/MS assay (AbsoluteIDQ 180 kit, Biocrates, Innsbruck, Austria) was applied to serum samples stored at −80 °C for the metabolomics analysis. The serum samples were processed in strict accordance with the instructions provided by the manufacturer. After the addition of 10 µL of the supplied internal standard solution to each well of the 96-well extraction plate, 10 µL of each serum sample was added to the appropriate well. The plate was then dried under a gentle stream of nitrogen. The samples were derivatized with phenylisothiocyanate and then eluted with 5 mM ammonium acetate in methanol. Samples were diluted with either 40% methanol in water for the LC-MS/MS analysis (15:1) or running solvent (a proprietary mixture provided by Biocrates) for FIA-MS/MS (20:1).

The analysis of amino acids and biogenic amines were resolved and quantified by LC-MS/MS using scheduled multiple reaction monitoring. Separation of amino acids and biogenic amines was performed using an Agilent 1260 Infinity High-Performance Liquid Chromatography system (Agilent Technologies Inc., Santa Clara, CA, USA) with a Waters Acquity 2.1 mm ×50 mm 1.7 µm BEH C18 column (Waters, Milford, MA, USA) fitted with a Waters Acquity BEH C18 1.7 µm Vanguard guard column. Analytes were separated using a gradient from 0.1% formic acid in water to 0.1% formic acid in acetonitrile. The total liquid chromatography analysis time was approximately 12 minutes per sample. Subsequent FIA-MS/MS was performed to analyze acylcarnitines, glycerophospholipids, and hexose, with a total analysis time of around 3 minutes per sample. Using electrospray ionization, samples for both liquid chromatography and flow injection analysis were introduced into a QTRAP 5500 mass spectrometer (SCIEX, Woodlands Central, Singapore) operating in multiple reaction monitoring mode. The measurements were made in a 96-well format. Seven calibration standards and three quality control samples were integrated into the plate. The LC-MS/MS data were imported into Sciex application Analyst™ for peak integration, calibration, and concentration calculations. The LC-MS/MS data from Analyst™ and FIA-MS/MS data were analyzed using a MetIDQ™ software (Biocrates).

This strategy allowed simultaneous quantification of 187 metabolites (42 amino acids and biogenic amines, 40 acylcarnitines, 90 glycerophospholipids, 14 sphingomyelins, and 1 monosaccharide). Seven metabolites were not included in the analysis because their levels were lower than the limit of detection.

### Statistical analysis

To assess population clustering and the parameters responsible for the distinction among groups, multivariate analyses were conducted both in a non-supervised method (PCA) and in a supervised method (PCA-DA), using the MarkerView (version 1.2.1, SCIEX) program with data normalized by the Pareto method. The mean values of the variables among patients with active TB and the other groups were then compared by Student’s t-test, and the false discovery rate adjusted p-values (q-values) were calculated to generate a volcano plot. After the selection of the key metabolites from those analyses, the median values of the metabolites among the patient groups were compared using Kruskal-Wallis or Mann-Whitney U tests using GraphPad Prism version 6.00 for Windows (GraphPad Software, La Jolla, CA, USA). ROC analysis was used to test the diagnostic utility of the metabolites in differentiating patients with active TB from those in the other groups using a R package ‘fbroc’ version 0.4.1 (Erik Peter, 2019). Finally, for the comparison of median values of the metabolites among the subgroups of patients with active TB, the patients were divided into two sub-groups according to the degree of risk burden (low or mid/high) and pulmonary extent (<1/3 or ≥1/3 of the total lung area), and Mann-Whitney U tests were conducted. We considered p-values <0.05 to be statistically significant.

## Supplementary information

Supplementary Information.

## Data Availability

All data used in this study are available at Harvard Dataverse (10.7910/DVN/PIMLZM).
