# Supplementary material for: Identification of serum biomarkers for active pulmonary tuberculosis using a targeted metabolomics approach
Source: Sci Rep. 2020 Mar 2;10:3825. doi: 10.1038/s41598-020-60669-0 (PMC7052258; doi:10.1038/s41598-020-60669-0)
Supplement: Supplementary file 1 — Supplementary Information. [file 41598_2020_60669_MOESM1_ESM.pdf]

Identification of serum biomarkers for active pulmonary tuberculosis using a targeted metabolomics approach

Yonggeun Cho[1†], Youngmok Park[2†], Bora Sim[3,4], Jungho Kim[3,4,5], Hyejon Lee[3,4], Sang-Nae Cho[3,4], Young Ae Kang[2,4*], Sang-Guk Lee[1*]

[1]Department of Laboratory Medicine, Severance Hospital, Yonsei University College of Medicine, Seoul, Republic of Korea

[2]Division of Pulmonology, Department of Internal Medicine, Severance Hospital, Yonsei University College of Medicine, Seoul, Republic of Korea

[3]Department of Microbiology, Yonsei University College of Medicine, Seoul, Republic of Korea

[4]Institute of Immunology and Immunological Diseases, Yonsei University College of Medicine, Seoul, Republic of Korea

[5]Department of Clinical Laboratory Science, College of Health Sciences, Catholic University of Pusan, Busan, Republic of Korea

SUPPLEMENTARY TABLES

Supplementary Table S1. List of the metabolites belonging to the significant area on the volcano plot of the 180 metabolites derived from the comparison between patients with active tuberculosis and those in the other groups (i.e., patients with latent tuberculosis and healthy controls).

Analyte	Fold change	FDR adjusted P-value[a]	AUC [95% CI][b]
Glutamine	0.2782	6.6708.E-18	1.0000 [NA]
Phosphatidylcholine diacyl C38:6	0.3101	2.3108.E-15	0.9871 [0.9633-1.0000]
Methionine	0.2066	1.0609.E-14	0.9916 [0.9717-1.0000]
Phosphatidylcholine diacyl C38:5	0.3844	6.0894.E-14	0.9802 [0.9325-1.0000]
Phosphatidylcholine diacyl C36:4	0.4917	5.3205.E-13	0.9861 [0.9583-1.0000]
Phosphatidylcholine diacyl C40:6	0.3527	2.6854.E-12	0.9643 [0.9147-0.9970]
Phosphatidylcholine acyl-alkyl C38:6	0.4114	9.7688.E-12	0.9544 [0.9018-0.9881]
Phosphatidylcholine acyl-alkyl C40:3	13.6965	6.6526.E-11	1.0000 [NA]
Phosphatidylcholine acyl-alkyl C38:3	8.0093	1.5535.E-10	1.0000 [NA]
Phosphatidylcholine diacyl C38:4	0.5336	2.9400.E-10	0.9583 [0.9087-0.9921]
Glutamate	3.6960	5.8219.E-10	0.9980 [0.9901-1.0000]
Phosphatidylcholine acyl-alkyl C40:4	4.2297	1.8340.E-09	1.0000 [NA]
Aspartate	2.0031	2.7802.E-09	0.9732 [0.9365-0.996]
Phosphatidylcholine acyl-alkyl C38:5	0.5436	3.2392.E-09	0.9315 [0.869-0.9802]
Phosphatidylcholine acyl-alkyl C38:1	26.5236	3.5483.E-09	1.0000 [NA]
Phosphatidylcholine acyl-alkyl C36:5	0.5201	8.7250.E-09	0.9177 [0.8413-0.9722]
Phosphatidylcholine acyl-alkyl C36:1	7.0018	1.1227.E-08	1.0000 [NA]

Phosphatidylcholine diacyl C26:0	2.1633	1.2126.E-08	0.9990 [0.994-1.0000]
Phosphatidylcholine diacyl C24:0	5.1939	1.2126.E-08	0.9990 [0.994-1.0000]
Phosphatidylcholine acyl-alkyl C42:1	6.5630	1.2126.E-08	1.0000 [NA]
Phosphatidylcholine acyl-alkyl C38:2	10.3047	1.2126.E-08	1.0000 [NA]
Phosphatidylcholine diacyl C34:4	0.5827	1.4150.E-08	0.8958 [0.8046-0.9643]
Phosphatidylcholine acyl-alkyl C30:2	5.8348	1.5119.E-08	1.0000 [NA]
Phosphatidylcholine diacyl C40:2	14.7802	1.5119.E-08	1.0000 [NA]
Phosphatidylcholine acyl-alkyl C40:5	4.4096	1.5718.E-08	0.9990 [0.9940-1.0000]
Asparagine	0.6585	2.0553.E-08	0.9325 [0.8631-0.9851]
Phosphatidylcholine acyl-alkyl C42:4	3.0855	2.2094.E-08	0.9911 [0.9683-1.0000]
Phosphatidylcholine diacyl C38:1	4.9007	2.2094.E-08	1.0000 [NA]
Phosphatidylcholine diacyl C40:5	0.5454	2.3281.E-08	0.9137 [0.8294-0.9802]
Phosphatidylcholine acyl-alkyl C40:2	4.5435	2.4123.E-08	1.0000 [NA]
Phosphatidylcholine diacyl C36:5	0.3238	2.5518.E-08	0.9395 [0.8810-0.9861]
lysoPhosphatidylcholine acyl C26:0	6.4653	2.5672.E-08	1.0000 [NA]
Phosphatidylcholine diacyl C42:4	11.9750	4.5463.E-08	1.0000 [NA]
Serotonin	0.5727	5.1076.E-08	0.8710 [0.7807-0.9420]
lysoPhosphatidylcholine acyl C24:0	3.7131	5.1076.E-08	0.9990 [0.9940-1.0000]
Phosphatidylcholine diacyl C42:2	5.0329	5.1076.E-08	1.0000 [NA]
Acetyl-L-carnitine	0.4849	5.3150.E-08	0.8998 [0.8085-0.9702]
lysoPhosphatidylcholine acyl C28:0	4.9381	5.6937.E-08	1.0000 [NA]
Methionine sulfoxide	14.8313	6.4080.E-08	1.0000 [NA]
Phosphatidylcholine diacyl C36:3	0.6959	7.7201.E-08	0.8998 [0.8135-0.9692]
lysoPhosphatidylcholine acyl C28:1	4.0044	7.7201.E-08	1.0000 [NA]
lysoPhosphatidylcholine acyl C26:1	6.3098	7.7201.E-08	1.0000 [NA]

Phosphatidylcholine diacyl C42:5	3.3058	7.9581.E-08	0.9980 [0.9911-1.0000]
Phosphatidylcholine acyl-alkyl C42:3	4.3210	8.4480.E-08	1.0000 [NA]
Phosphatidylcholine acyl-alkyl C42:2	3.9342	8.6238.E-08	1.0000 [NA]
Phosphatidylcholine diacyl C42:0	2.2734	1.0864.E-07	0.9851 [0.9613-1.0000]
Phosphatidylcholine acyl-alkyl C42:5	3.0749	1.0864.E-07	1.0000 [NA]
Phosphatidylcholine diacyl C40:3	8.2331	1.3747.E-07	1.0000 [NA]
Phosphatidylcholine diacyl C42:1	3.1572	1.6326.E-07	0.9990 [0.9940-1.0000]
Phosphatidylcholine diacyl C36:0	2.2926	2.0462.E-07	0.9633 [0.9177-0.9921]
Phosphatidylcholine acyl-alkyl C40:1	2.6852	2.0462.E-07	1.0000 [NA]
Phosphatidylcholine acyl-alkyl C44:3	6.6269	2.0462.E-07	1.0000 [NA]
Phosphatidylcholine diacyl C40:1	4.1661	3.9455.E-07	1.0000 [NA]
Phosphatidylcholine acyl-alkyl C44:4	2.3703	4.1959.E-07	0.9980 [0.9911-1.0000]
Phosphatidylcholine acyl-alkyl C30:1	2.5463	4.2635.E-07	0.9356 [0.8701-0.9816]
Phosphatidylcholine acyl-alkyl C36:4	0.6188	5.1767.E-07	0.8482 [0.7381-0.9365]
Phosphatidylcholine acyl-alkyl C42:0	1.8159	1.0670.E-06	0.9871 [0.9643-1.0000]
Butyryl-L-carnitine	0.6455	1.8492.E-06	0.8447 [0.7296-0.9350]
Phosphatidylcholine diacyl C40:4	2.7934	3.4390.E-06	0.9921 [0.9742-1.0000]
Phosphatidylcholine acyl-alkyl C36:2	1.6180	5.8268.E-06	0.8899 [0.8006-0.9633]

[a] P-values were calculated using Mann-Whitney U tests.

[b] The AUCs of receiver characteristic analysis to predict patients with active tuberculosis were presented with their 95% CI computed by 2000 stratified bootstrap replicates. For the metabolite or ratio with the AUC value of 1, CI was not presented to avoid misunderstanding.

Abbreviations: FDR, false discovery rate; AUC, area under the curve; CI, confidence interval; NA, not available

Supplementary Table S2. List of the metabolites belonging to the significant area on the volcano plot of 76 non-lipid metabolites (amino acids, biogenic amines, acyl-carnitines, and hexose) derived from the comparison between patients with active tuberculosis and those in the other groups (i.e., patients with latent tuberculosis and healthy controls).

**Analyte**	**Fold change**	**FDR adjusted P-value[a]**
Glutamine	0.2782	1.2971.E-18
Methionine	0.2066	3.0942.E-15
Glutamate	3.6960	4.1508.E-10
Aspartate	2.0031	1.7569.E-09
Asparagine	0.6585	2.0781.E-08
Serotonin	0.5727	5.9589.E-08
Methionine sulfoxide	14.8313	6.9420.E-08
Acetyl-L-carnitine	0.4849	1.1862.E-07
Butyryl-L-carnitine	0.6455	5.1184.E-06

[a] P-values were calculated using Mann-Whitney U tests.

Abbreviations: FDR, false discovery rate

SUPPLEMENTARY FIGURES AND FIGURE LEGENDS

Supplementary Figure S1. Principal component analysis (PCA) score and loading plot of the metabolites measured in patients with active tuberculosis, those with latent tuberculosis, and healthy controls.

(A) In the score plot, each dot represents an individual and is colored in accordance with the embedded legends. (B) The loadings for the two principal components (PC1 and PC2) are depicted. The metabolites located distant from the origin are labeled with their chemical names. Abbreviations: Glu, glutamate; Gln, glutamine; PC, phosphatidylcholine

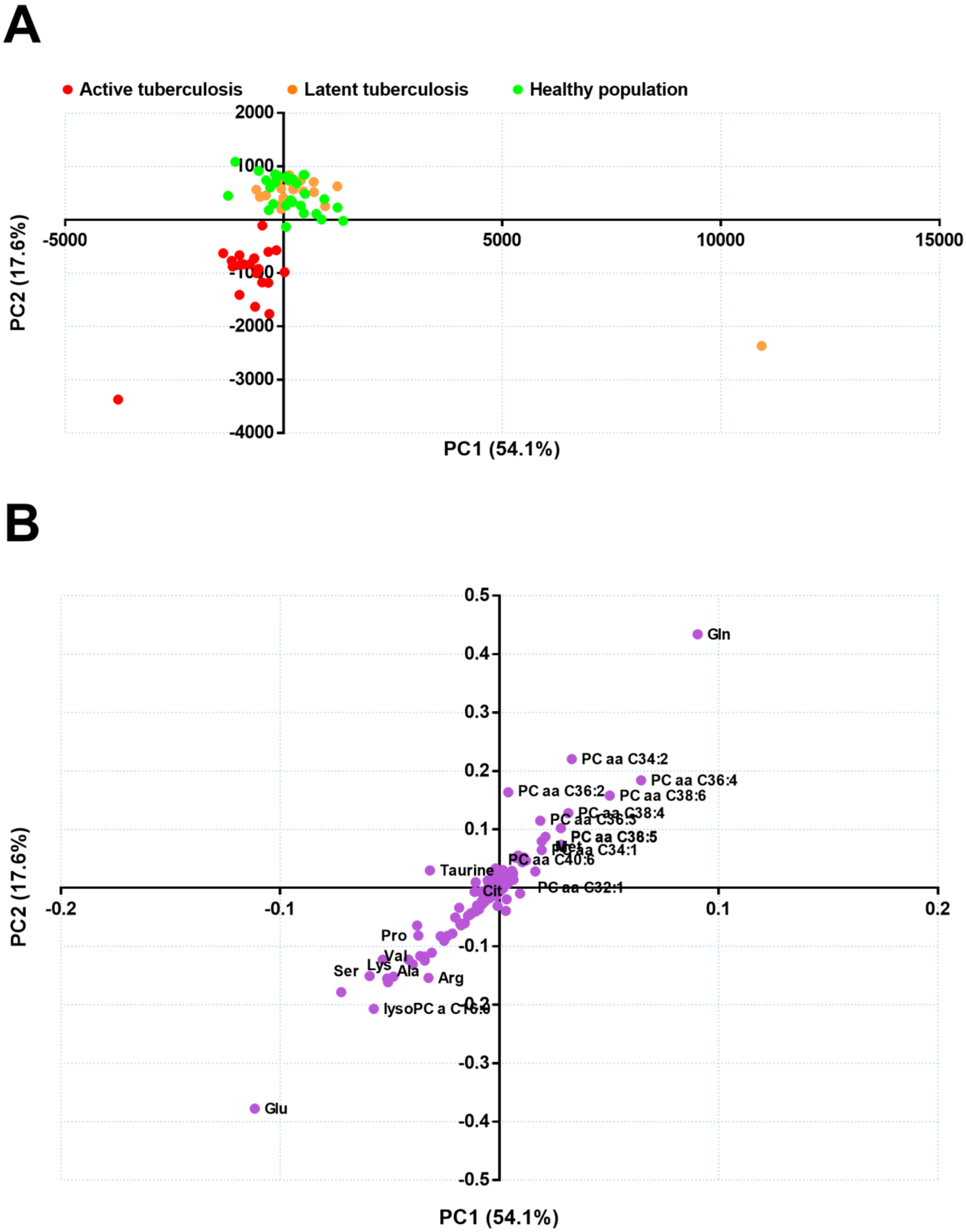
A
Active tuberculosis
Latent tuberculosis
Healthy population
PC2 (17.6%)
PC1 (54.1%)
B
Gln
PC aa C34:2
PC aa C36:4
PC aa C36:2
PC aa C38:6
PC aa C38:4
PC aa C36:3
PC aa C36:5
Met
PC aa C34:1
PC aa C40:6
Taurine
Cit
PC aa C32:1
Pro
Val
Lys
Ser
Ala
Arg
lysoPC a C16:0
Glu
PC2 (17.6%)
PC1 (54.1%)

Supplementary Figure S2. Volcano plot of all 180 metabolites derived from the comparison between patients with active tuberculosis and the others (patients with latent tuberculosis and healthy population).

A total of 76 non-lipid metabolites (amino acids, biogenic amines, acyl-carnitines, and hexose) are shown as red dots, and the other 104 lipid metabolites (lysophosphatidylcholines, phosphatidylcholines, sphingomyelins) are shown as grey dots.

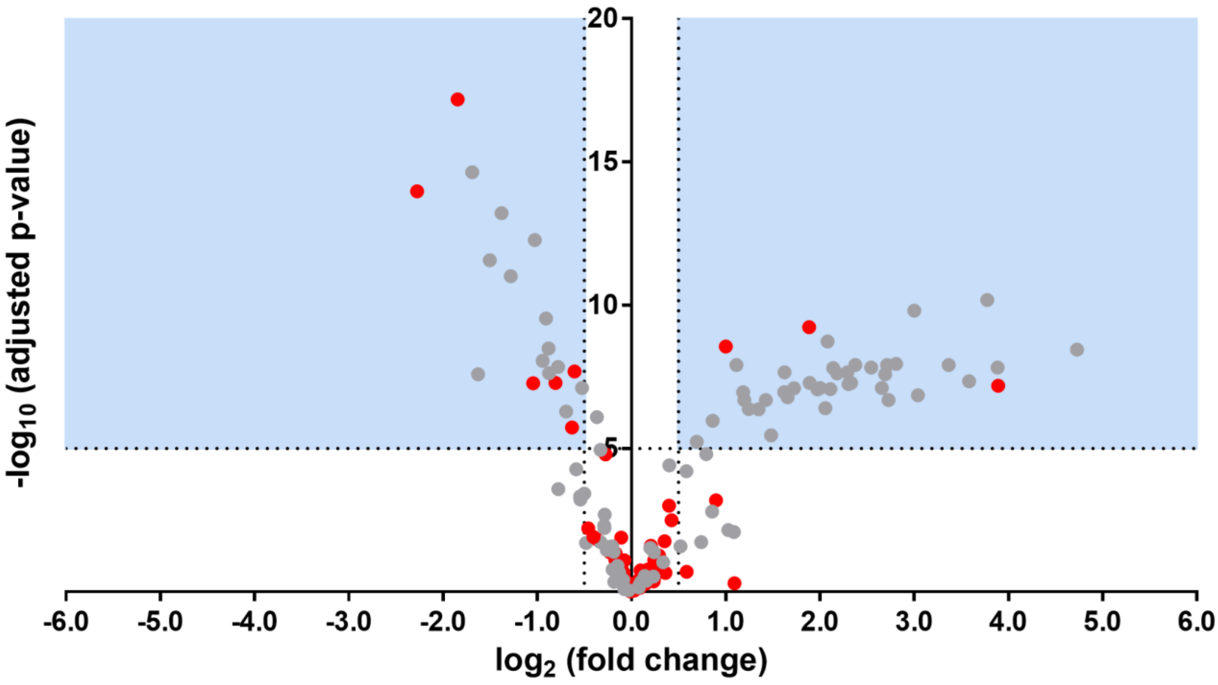

Supplementary Figure S3. The concentrations of representative lipid metabolites among study populations. The median values of metabolites were compared among the healthy population, patients with latent tuberculosis, and patients with active tuberculosis using Kruskal-Wallis tests.

Abbreviations: PC, phosphatidylcholine; LysoPC, lysophosphatidylcholine; aa, diacyl; ae, acyl-alkyl; a, acyl

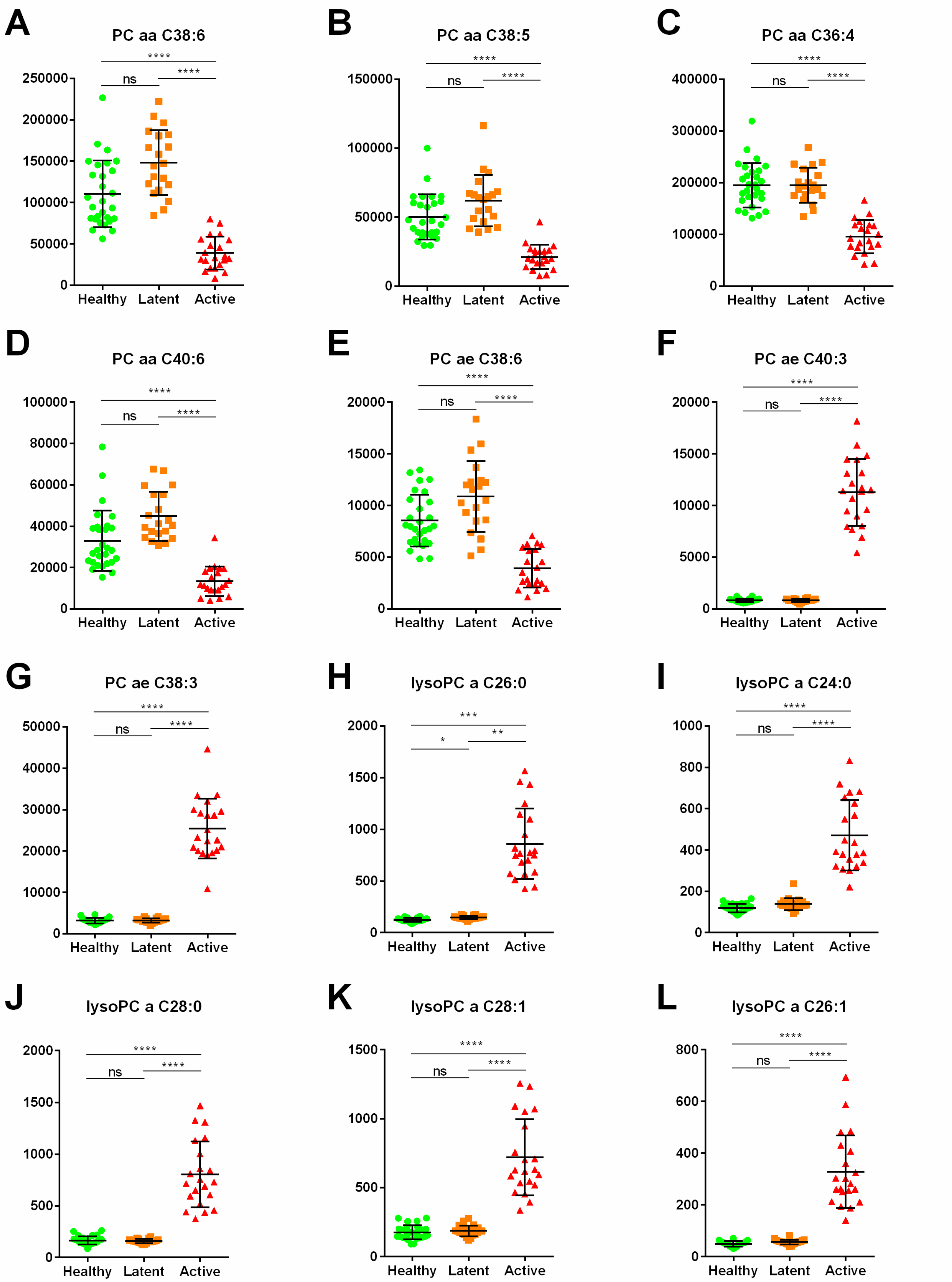
A
PC aa C38:6
****
ns
****
250000
200000
150000
100000
50000
0
Healthy
Latent
Active
B
PC aa C38:5
****
ns
****
150000
100000
50000
0
Healthy
Latent
Active
C
PC aa C36:4
****
ns
****
400000
300000
200000
100000
0
Healthy
Latent
Active
D
PC aa C40:6
****
ns
****
100000
80000
60000
40000
20000
0
Healthy
Latent
Active
E
PC ae C38:6
****
ns
****
20000
15000
10000
5000
0
Healthy
Latent
Active
F
PC ae C40:3
****
ns
****
20000
15000
10000
5000
0
Healthy
Latent
Active
G
PC ae C38:3
****
ns
****
50000
40000
30000
20000
10000
0
Healthy
Latent
Active
H
lysoPC a C26:0
***
*
**
2000
1500
1000
500
0
Healthy
Latent
Active
I
lysoPC a C24:0
****
ns
****
1000
800
600
400
200
0
Healthy
Latent
Active
J
lysoPC a C28:0
****
ns
****
2000
1500
1000
500
0
Healthy
Latent
Active
K
lysoPC a C28:1
****
ns
****
1500
1000
500
0
Healthy
Latent
Active
L
lysoPC a C26:1
****
ns
****
800
600
400
200
0
Healthy
Latent
Active

Supplementary Figure S4. ROC curves of representative lipid metabolites for the differentiation of patients with active tuberculosis from those in the other groups, including patients with latent tuberculosis infection and healthy controls.

Blue lines indicate ROC curves, and royal blue regions show the 95% CI of the curve computed with 2000 bootstrap replicates. The AUC, followed by its 95% CI in brackets, were also denoted over the bottom side of each figure. For the metabolite with an AUC value of 1, CI was not presented to avoid misunderstanding.

Abbreviations: TPR, true positive rate; FPR, false positive rate; PC, phosphatidylcholine; LysoPC, lysophosphatidylcholine; aa, diacyl; ae, acyl-alkyl; a, acyl; NA, not available; ROC, receiver operating characteristic; CI, confidence interval; AUC, area under the curve

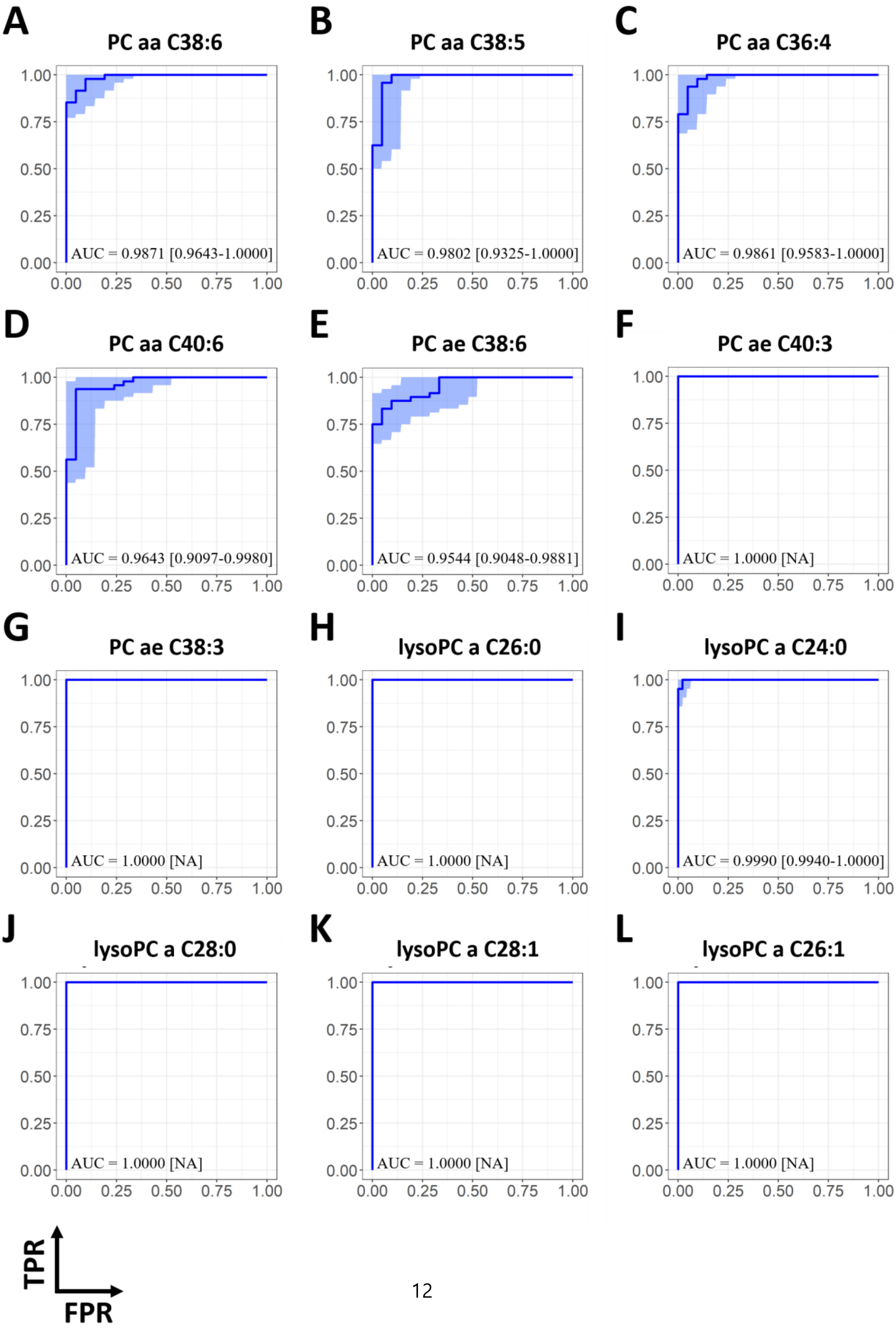
A
PC aa C38:6
AUC = 0.9871 [0.9643-1.0000]
B
PC aa C38:5
AUC = 0.9802 [0.9325-1.0000]
C
PC aa C36:4
AUC = 0.9861 [0.9583-1.0000]
D
PC aa C40:6
AUC = 0.9643 [0.9097-0.9980]
E
PC ae C38:6
AUC = 0.9544 [0.9048-0.9881]
F
PC ae C40:3
AUC = 1.0000 [NA]
G
PC ae C38:3
AUC = 1.0000 [NA]
H
lysoPC a C26:0
AUC = 1.0000 [NA]
I
lysoPC a C24:0
AUC = 0.9990 [0.9940-1.0000]
J
lysoPC a C28:0
AUC = 1.0000 [NA]
K
lysoPC a C28:1
AUC = 1.0000 [NA]
L
lysoPC a C26:1
AUC = 1.0000 [NA]
TPR
FPR

Supplementary Figure S5. The levels or ratios of selected metabolites among patients with active tuberculosis harboring different risks.

The patients with active tuberculosis on into two subgroups according to their degree of risk burden (low or mid/high). The concentrations of selected metabolites and their ratios were compared using Mann-Whitney U tests. Note that the figures representing Glu/Gln and MetSO/Met ratio are on a logarithmic scale (C and F), while the others are on a linear scale.

Abbreviations: Glu, glutamate; Gln, glutamine; Met, methionine; MetSO, sulfoxy methionine; Kyn, kynurenine; Trp, tryptophan

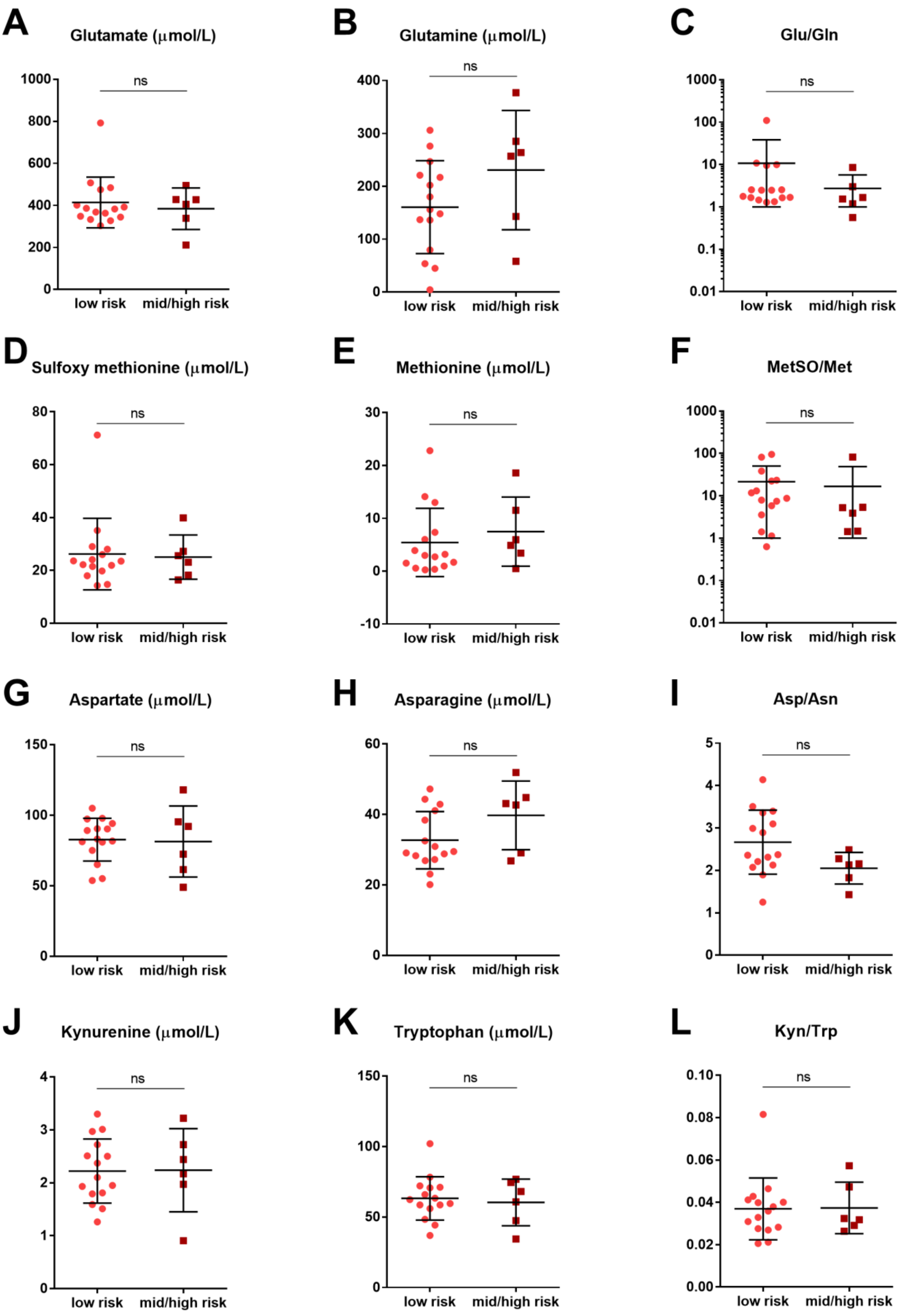
A
Glutamate (μmol/L)
1000
800
600
400
200
0
ns
low risk
mid/high risk
B
Glutamine (μmol/L)
400
300
200
100
0
ns
low risk
mid/high risk
C
Glu/Gln
1000
100
10
1
0.1
0.01
ns
low risk
mid/high risk
D
Sulfoxy methionine (μmol/L)
80
60
40
20
0
ns
low risk
mid/high risk
E
Methionine (μmol/L)
30
20
10
0
-10
ns
low risk
mid/high risk
F
MetSO/Met
1000
100
10
1
0.1
0.01
ns
low risk
mid/high risk
G
Aspartate (μmol/L)
150
100
50
0
ns
low risk
mid/high risk
H
Asparagine (μmol/L)
60
40
20
0
ns
low risk
mid/high risk
I
Asp/Asn
5
4
3
2
1
0
ns
low risk
mid/high risk
J
Kynurenine (μmol/L)
4
3
2
1
0
ns
low risk
mid/high risk
K
Tryptophan (μmol/L)
150
100
50
0
ns
low risk
mid/high risk
L
Kyn/Trp
0.10
0.08
0.06
0.04
0.02
0.00
ns
low risk
mid/high risk

Supplementary Figure S6. The levels or ratios of selected metabolites among patients with active tuberculosis with different extents of pulmonary involvement.

The patients with active tuberculosis were divided into two sub-groups according to the extent of pulmonary involvement of their TB ($< 1/3$ or $\geq 1/3$ of the total lung area). The concentrations of selected metabolites and their ratios were compared using Mann-Whitney U tests. Note that figures representing the Glu/Gln or MetSO/Met ratio are on a logarithmic scale (C and F), while the others are on a linear scale.

Abbreviations: Glu, glutamate; Gln, glutamine; Met, methionine; MetSO, sulfoxy methionine; Kyn, kynurenine; Trp, tryptophan

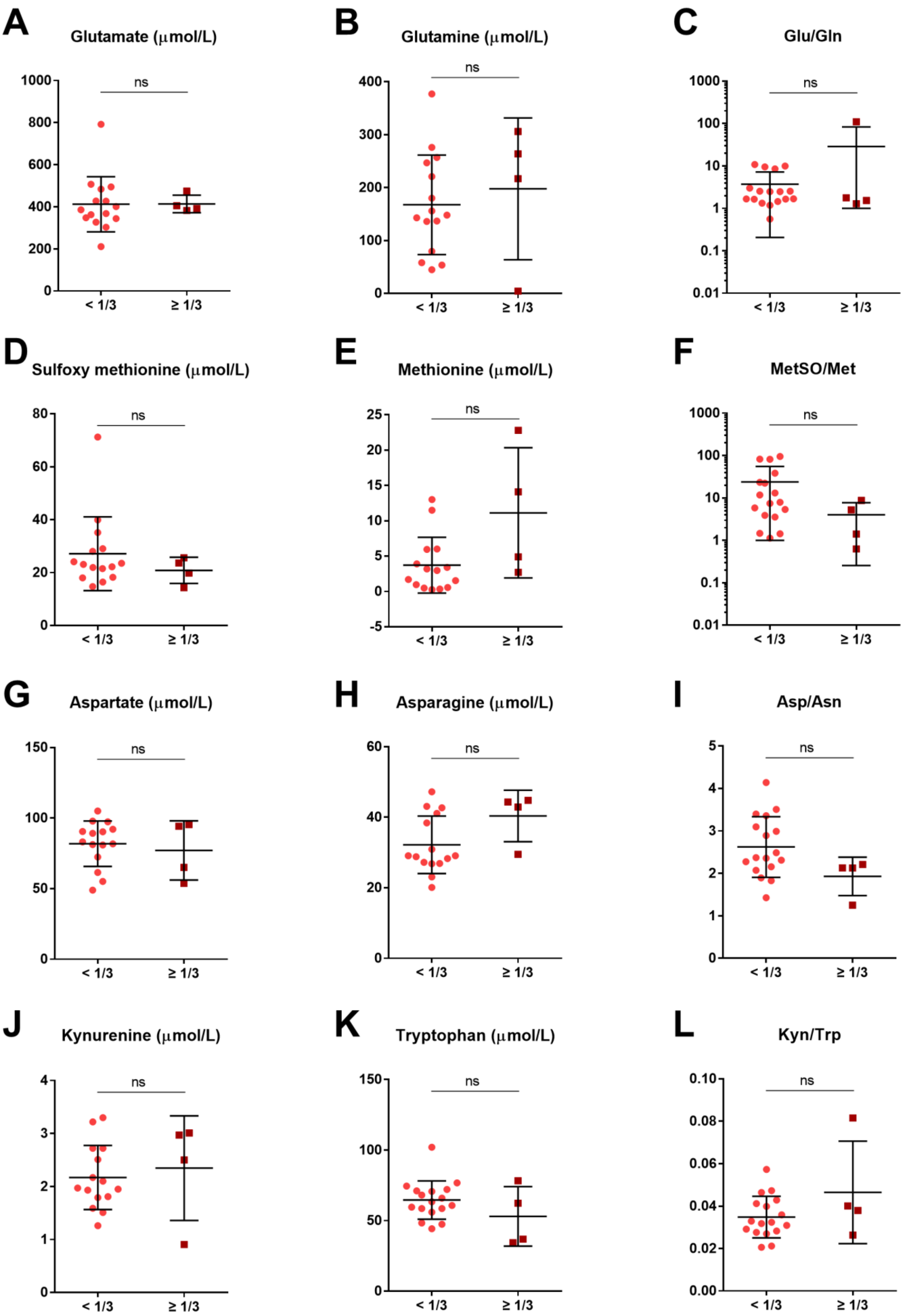
A
Glutamate (μmol/L)
ns
< 1/3
≥ 1/3
B
Glutamine (μmol/L)
ns
< 1/3
≥ 1/3
C
Glu/Gln
ns
< 1/3
≥ 1/3
D
Sulfoxy methionine (μmol/L)
ns
< 1/3
≥ 1/3
E
Methionine (μmol/L)
ns
< 1/3
≥ 1/3
F
MetSO/Met
ns
< 1/3
≥ 1/3
G
Aspartate (μmol/L)
ns
< 1/3
≥ 1/3
H
Asparagine (μmol/L)
ns
< 1/3
≥ 1/3
I
Asp/Asn
ns
< 1/3
≥ 1/3
J
Kynurenine (μmol/L)
ns
< 1/3
≥ 1/3
K
Tryptophan (μmol/L)
ns
< 1/3
≥ 1/3
L
Kyn/Trp
ns
< 1/3
≥ 1/3

Supplementary Figure S7. The levels or ratios of selected metabolites among the study population by Wiener et al. (2012).

The median values of selected metabolites and their ratios were compared among the healthy population, patients with latent tuberculosis, and patients with active tuberculosis using Kruskal-Wallis tests. Note that the figure representing Glu/Gln is on a logarithmic scale (C), while the others are on a linear scale. The levels represent relative abundances of over 400 small compounds obtained by mass spectrometry. Although sulfoxy methionine was not measured in the study, figure (D) and (F) were drawn intentionally left blank for the ease of comparison with Figure 3.

Abbreviations: Glu, glutamate; Gln, glutamine; Met, methionine; MetSO, sulfoxy methionine; Asp, aspartate; Asn, asparagine; Kyn, kynurenine; Trp, tryptophan

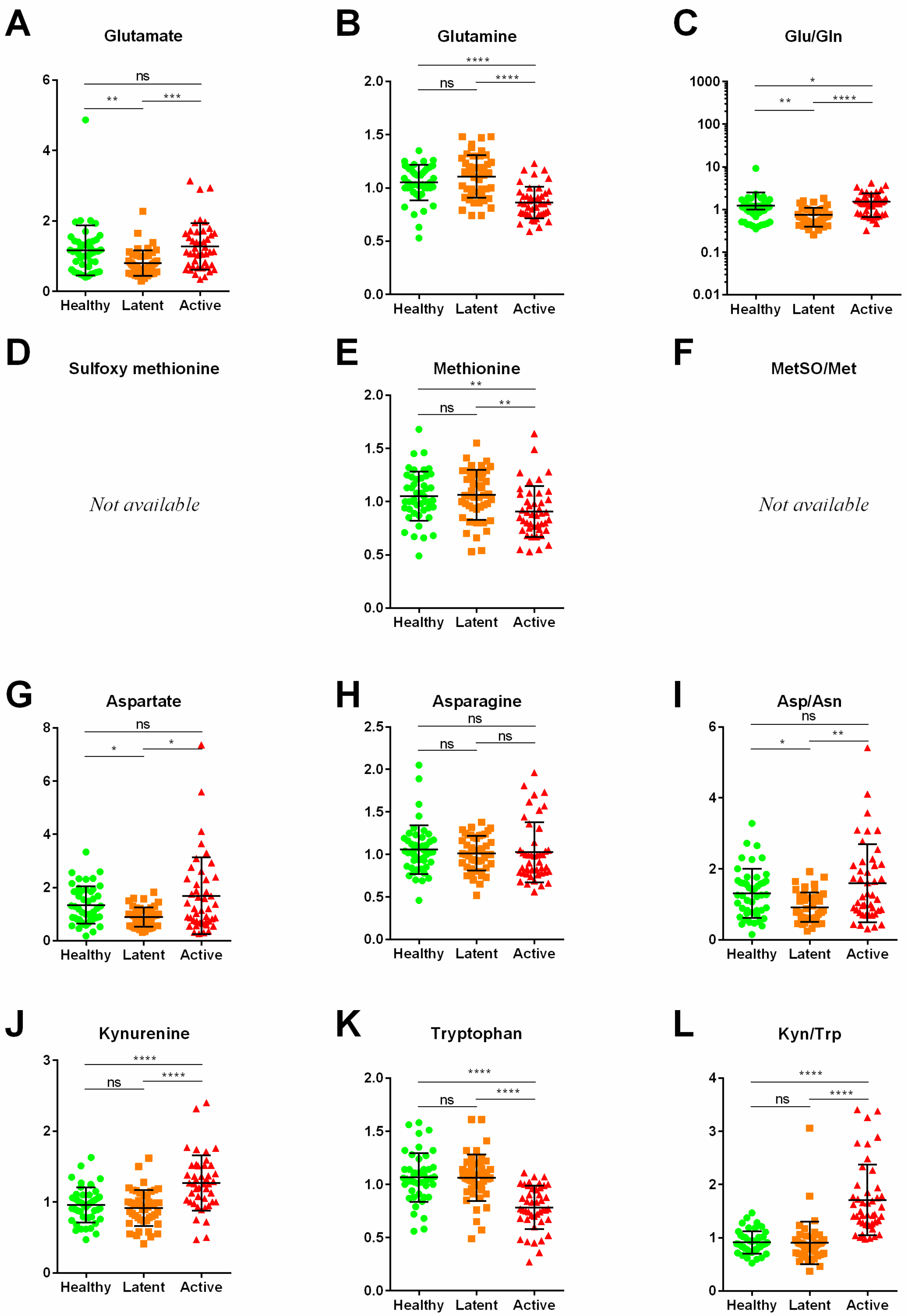
A
Glutamate
ns
**
***
Healthy
Latent
Active
B
Glutamine
****
ns
****
Healthy
Latent
Active
C
Glu/Gln
*
**
****
Healthy
Latent
Active
D
Sulfoxy methionine
Not available
E
Methionine
**
ns
**
Healthy
Latent
Active
F
MetSO/Met
Not available
G
Aspartate
ns
*
*
Healthy
Latent
Active
H
Asparagine
ns
ns
ns
Healthy
Latent
Active
I
Asp/Asn
ns
*
**
Healthy
Latent
Active
J
Kynurenine
****
ns
****
Healthy
Latent
Active
K
Tryptophan
****
ns
****
Healthy
Latent
Active
L
Kyn/Trp
****
ns
****
Healthy
Latent
Active

Supplementary Figure S8. ROC curves of the selected metabolites and their ratios for the differentiation of patients with active tuberculosis from those in the other groups, including patients with latent tuberculosis infection and healthy controls, from the study population by Weiner et al (2012).

Blue lines indicate ROC curves, and royal blue regions show the 95% CI of the curve computed with 2000 bootstrap replicates. The AUC, followed by its 95% CI in brackets, were also denoted over the bottom side of each figure. For the metabolite or ratio with AUC value of 1, CI was not presented to avoid misunderstanding.

Abbreviations: TPR, true positive rate; FPR, false positive rate; Glu, glutamate; Gln, glutamine; Met, methionine; MetSO, sulfoxy methionine; Asp, aspartate; Asn, asparagine; Kyn, kynurenine; Trp, tryptophan; ROC, receiver operating characteristic; CI, confidence interval; AUC, area under the curve

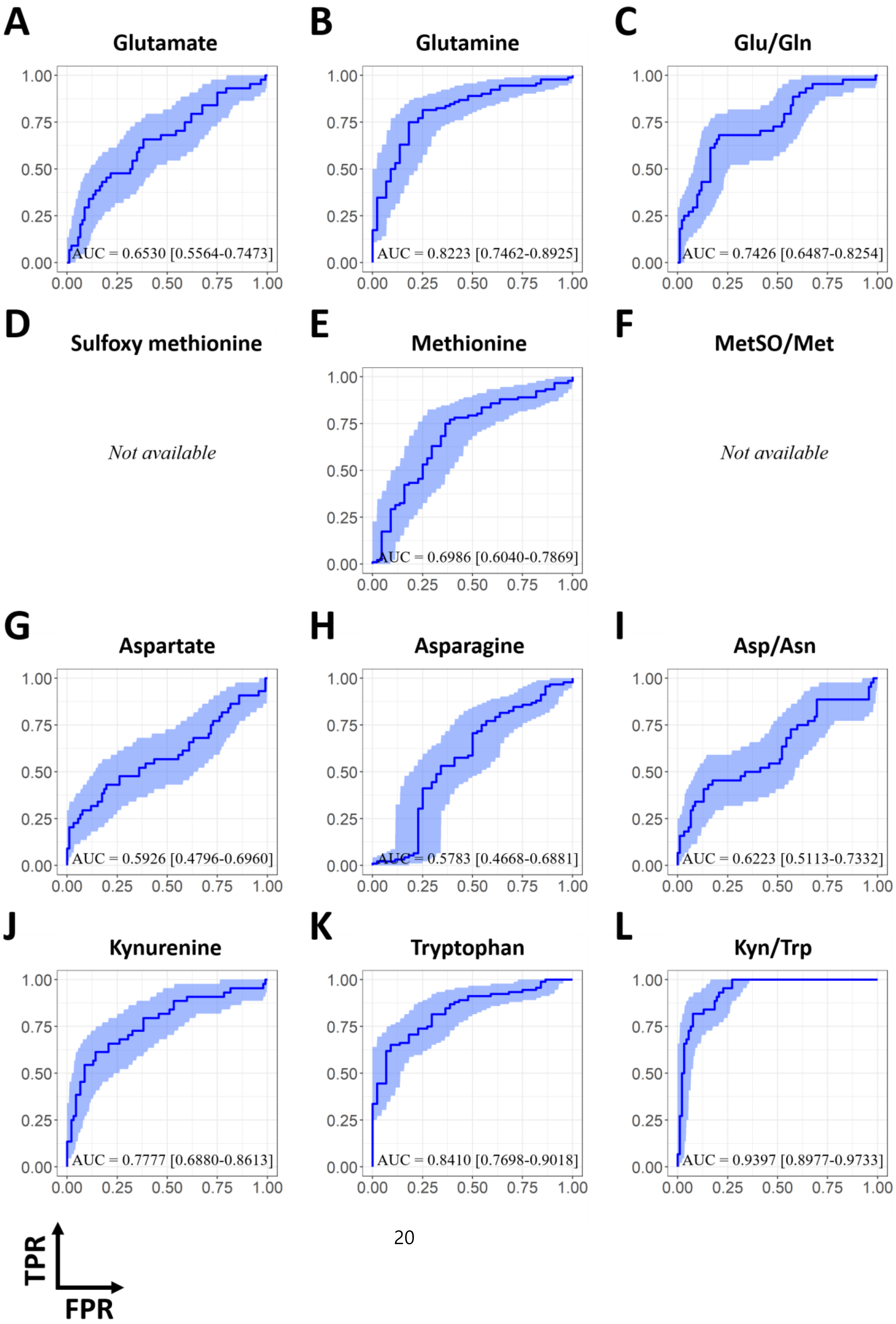
A
Glutamate
AUC = 0.6530 [0.5564-0.7473]
B
Glutamine
AUC = 0.8223 [0.7462-0.8925]
C
Glu/Gln
AUC = 0.7426 [0.6487-0.8254]
D
Sulfoxy methionine
Not available
E
Methionine
AUC = 0.6986 [0.6040-0.7869]
F
MetSO/Met
Not available
G
Aspartate
AUC = 0.5926 [0.4796-0.6960]
H
Asparagine
AUC = 0.5783 [0.4668-0.6881]
I
Asp/Asn
AUC = 0.6223 [0.5113-0.7332]
J
Kynurenine
AUC = 0.7777 [0.6880-0.8613]
K
Tryptophan
AUC = 0.8410 [0.7698-0.9018]
L
Kyn/Trp
AUC = 0.9397 [0.8977-0.9733]
TPR
FPR
